# Early uptake of doxycycline prophylaxis among MSM in Rome: Insights from a reference STI clinic

**DOI:** 10.1177/09564624251375877

**Published:** 2025-10-09

**Authors:** Maria Gabriella Donà, Mauro Zaccarelli, Massimo Giuliani, Christof Stingone, Laura Gianserra, Francesca Di Tullio, Eugenia Giuliani, Alessandra Latini

**Affiliations:** STI/HIV Unit, San Gallicano Dermatological Institute IRCCS, Rome, Italy

**Keywords:** Doxycycline, STI prophylaxis, MSM, PrEP, antimicrobial resistance

## Abstract

**Background:**

Prompt doxycycline use (200 mg taken 24–72 hours after sexual activity, i.e., post-exposure prophylaxis, Doxy-PEP), effectively reduces chlamydia and syphilis acquisition in men who have sex with men (MSM) and individuals experiencing recurrent STIs. Considering the IUSTI Europe position statement and recent guidelines from the CDC, this study investigated the use of doxycycline to prevent STIs among MSM attendees of an STI/HIV center in Rome, Italy.

**Methods:**

From June 2024 to February 2025, an anonymous self-administered questionnaire was used to collect data on sexual behaviors, HIV status, HIV pre-exposure prophylaxis (PrEP), STI history, recreational substances, and doxycycline PrEP/PEP use. Logistic regression analyzed associations between Doxy-PEP/PrEP use and behavioral/clinical factors.

**Results:**

Two hundred and ninety MSM were enrolled. Among them, 9.3% reported using Doxy-PEP/PrEP, with nearly half accessing it without medical supervision. Multivariable analysis showed significant associations between doxycycline use and having ≥20 sexual partners, erectile drug use, and condomless anal sex.

**Conclusions:**

While doxycycline remains an uncommon choice for STI prevention among our attendees, its usage is linked to individuals engaging in sex with multiple partners. These findings highlight the urgency of national guidelines to address informal prophylactic antibiotic use and ensure supervised STI prevention strategies among individuals at elevated risk for bacterial STIs.

## Introduction

According to the epidemiological reports on sexually transmitted infections (STIs) of the European Centre for Disease Prevention and Control (ECDC), STIs continue to increase.^1–3^ Over 41,000 confirmed cases of syphilis, 96,000 of gonorrhea and 230,000 of chlamydia were observed in 2023 with a 100%, 321% and 13% increase, respectively, compared to 2014.^1–3^ The year 2022 and 2023 saw the highest number of chlamydia and gonorrhea cases, respectively, since the start of STI surveillance in Europe.^2,3^ Significant increases have been observed in men who have sex with men (MSM), particularly for syphilis. In 2023, over 70% of the cases were diagnosed in MSM.^1^ Taken together, these observations underscore the urgent need for effective STI preventive strategies.

Doxycycline, a broad-spectrum tetracycline-class antibiotic that blocks bacterial protein synthesis, has long-standing clinical applications in treating bacterial infections such as acne, respiratory tract infections, and STIs. Recently, interest in its use for chemoprophylaxis of STIs has been growing. In particular, doxycycline has been explored for post-exposure prophylaxis (PEP) and/or pre-exposure prophylaxis (PrEP) targeting bacterial STIs among gay, bisexual and other MSM, transgender women (TW), and cisgender women living with HIV or taking HIV-PrEP. Randomized clinical trials have demonstrated that doxycycline, when administered shortly after condomless sex (24–72 hours), can reduce the incidence of STIs such as syphilis and chlamydia, particularly in MSM and transgender women (TW).^4–7^ In response to these findings, the Centers for Disease Control and Prevention (CDC) included Doxy-PEP in its 2024 guidelines for STI prevention in gay, bisexual, and other MSM, and TW with a history of at least one bacterial STI (specifically syphilis, chlamydia, or gonorrhea) during the past 12 months.^8^ However, adoption in Europe remains limited. Regulatory bodies and professional societies have expressed concern about long-term safety, ecological impact, and the potential for inducing antimicrobial resistance.^9^ The IUSTI Europe Position Statement on use of DoxyPEP currently recommends doxycycline prophylaxis only for individuals who are most likely to benefit from this intervention and under appropriate monitoring.^10^ More recently, the British Association for Sexual Health and HIV (BASHH) has developed evidence-based recommendations for the use of Doxy-PEP for the prevention of syphilis in gay, bisexual and other MSM, as well as TW, at elevated risk of acquiring syphilis.^11^ A single dose of 200 mg of doxycycline within 24–72 hours after sex is recommended. STI surveillance strategies to monitor the impact of Doxy-PrEP/PEP and antibiotic stewardship are strongly emphasized in the German STI Society Position Statement on the use of doxycycline to prevent STIs.^12^

In light of the growing global interest and policy divergence, we aimed to investigate the prevalence and correlates of doxycycline prophylaxis among MSM attending a high-volume STI/HIV reference center in Rome, Italy. Our objective was to explore behavioral and clinical patterns associated with Doxy-PEP and Doxy-PrEP use, including prescribing practices and informal access.

## Methods

Between June 2024 and February 2025, MSM attending the STI/HIV Center of the San Gallicano Dermatological Institute IRCCS (Rome, Italy) were invited to participate in the survey. The Center includes an STI clinic for the prevention, diagnosis and treatment of STIs as well as an HIV clinic for the treatment and care of people living with HIV (PLWH). The former is part of the Italian STI Surveillance System. The survey was conducted through an anonymous self-administered questionnaire designed to capture demographic data, sexual behavior during the previous year (e.g., number of partners, condom use, group sex), and use of digital platforms for partner seeking. Additionally, participants were asked to report their HIV status, HIV-PrEP use (if HIV-negative), and history of STIs. Information on substance use was also obtained, including erectile dysfunction agents (EDA) and chemsex-associated drugs such as crystal methamphetamine, GBL, and synthetic cathinones. A dedicated section focused on doxycycline use for STI prevention. Participants were asked whether they had used doxycycline in the past 12 months for either PEP or PrEP, and how they accessed it, i.e., via formal prescription or informal channels. Once informed about the survey, the attendees who agreed to participate were offered two options for self-compiling the questionnaire: they could either fill it online (via the *Pensa Rapido* web portal, managed by the Center), while at the Center or at their convenience, or in a paper version in person prior to their consultation.

Descriptive statistics were used to summarize the characteristics of the survey participants. Logistic regression models were used to investigate associations between doxycycline prophylaxis and behavioral/clinical variables. Variables that were significant (*p* < .05) in univariable models were included in the multivariable analysis. Age was also included as a covariate regardless of significance in univariable analysis. All statistical analyses were conducted using the SPSS software (version 27.0).

## Results

A total of 290 MSM completed the questionnaire (median age: 42 years, IQR: 32–52). Of these, 168 (57.9%) responded online and 122 (42.1%) completed the paper version. The characteristics of the respondents are shown in Table 1. PLWH accounted for 106 respondents (36.6%), all undergoing stable antiretroviral therapy. Forty-three individuals (14.8%) reported having 20 or more sexual partners in the previous year. Nearly one-third (90/290, 31.0%) engaged in group sex and 157 subjects (54.1%) reported inconsistent condom use during anal intercourse. The use of EDAs was reported by 67 individuals (23.1%), and 33 (11.4%) reported recent use of chemsex-related substances. Of the 290 participants, 223 (76.9%) used dating apps to search for sexual partners. Among the 184 HIV-negative MSM, 28 (15.2%) were taking HIV-PrEP at the time of the survey.

**Table 1.** Characteristics of the study population represented by 290 participants.

Variable	*n* (%)
STI history	228 (78.6)
MSMW	37 (12.8)
≥20 partners	43 (14.8)
Group sex	90 (31.0)
Condomless sex^a^	157 (54.1)
EDA use	67 (23.1)
Chems use^b^	33 (11.4)
Dating apps use	220 (76.9)
Doxycycline prophylaxis	
PrEP	7 (2.4)
PEP	20 (6.9)
PLWH	106 (36.6)

^a^In insertive or receptive anal sex.

^b^Chems included Chrystal Methamphetamine, Mephedrone, GBL, Ketamine, MDMA, Synthetic Cathinones, Cocaine/Crack.

Among the participants, 27 (9.3%) reported use of doxycycline for STI prophylaxis in the previous 12 months: 20 (6.9%) used it as PEP and 7 (2.4%) as PrEP. Notably, 15/27 (55.6%) users had received a formal medical prescription, 13 from infectious disease specialists and two from general practitioners. Other sources used to obtain the antibiotic included online platforms (8/27, 29.6%) and sexual partners (4/27, 14.9%).

Multivariable analysis showed that use of doxycycline for STI prophylaxis was significantly associated with having had 20 or more partners in the previous year [adjusted odds ratio (AOR): 2.86; 95% confidence interval (CI): 1.12–7.26; *p* = .03] and use of EDAs (AOR: 2.69; 95% CI: 1.12–6.45; *p* = .03) (Table 2). The association with inconsistent condom use was at the threshold of statistical significance (AOR: 2.92; 95% CI: 1.00–8.52; *p* = .05). No significant associations were found for age, use of chemsex-related substances and current HIV-PrEP use (adjusted by HIV-negative MSM), all significant at univariate analysis (data not shown).

**Table 2.** Univariable and multivariable logistic regression analysis to investigate association of doxycycline prophylaxis with selected covariates.

Variable	COR	95% CI	*p* Value	AOR	95% CI	*p* Value
Age (≥50 years)	0.97	0.77–1.22	0.78	0.90	0.69–1.18	0.45
STI history	3.63	0.84–15.79	0.09			
MSMW	1.21	0.39–3.73	0.74			
≥20 partners	**4.10**	**1.73–9.71**	**<0.01**	**2.86**	**1.12–7.26**	**0.03**
Group sex	1.90	0.85–4.24	0.12			
Condomless sex^a^	**4.17**	**1.53–11.35**	**<0.01**	2.92	1.00–8.52	0.05
Current HIV-PrEP use	**3.13**	**1.14–8.57**	**0.03**	2.35^b^	0.71–7.75	0.16
EDA use	**3.59**	**1.60–8.10**	**<0.01**	**2.69**	**1.10–6.37**	**0.03**
Chems use^c^	**4.01**	**1.59–10.09**	**<0.01**	1.99	0.71–5.50	0.19
Dating apps use	1.44	0.53–3.97	0.48			
PLWH	1.44	0.65–3.20	0.37			

COR: Crude odds ratio, AOR: Adjusted odds ratio; EDA: Erectile dysfunction agents. Statistical significance is highlighted in bold.

^a^In insertive or receptive anal sex.

^b^Adjusted by HIV status.

^c^Chems included Chrystal Methamphetamine, Mephedrone, GBL, Ketamine, MDMA, Synthetic Cathinones, Cocaine/Crack.

## Discussion

European and Italian data clearly indicate that bacterial STIs are on the rise and that syphilis particularly affects MSM.^1,13,14^ Of the 3093 syphilis cases observed in our Center during a 30-year period, approximately 65% were diagnosed in MSM,^13^ consistently with data from the Italian Surveillance System and ECDC.^1,14^ Between 2000 and 2021, a 23.2% increase in STI diagnoses in MSM has been reported in our country and, in the most recent years, MSM have experienced the greatest increases for all bacterial STIs.^14^

Despite international endorsement and robust clinical evidence, the use of doxycycline prophylaxis remains limited among our MSM attendees. Indeed, less than 10% of the respondents reported Doxy-PEP/PrEP use. Another Italian survey reported Doxy-PEP use in a similar proportion of subjects (8.8%), although it was conducted exclusively among HIV-PrEP users.^15^ Higher proportions of users have been reported in other European studies. The prevalence is around 15% among MSM in Netherlands, where Doxy-PEP is not formally recommended,^16^ and is even higher in an STI outpatient clinic in southwestern Germany, where 23% of the MSM attendees were Doxy-PEP and 6% Doxy-PrEP users.^17^ Doxy-PEP use has rapidly diffused in MSM communities in San Francisco, where over 19% reported its use just 1 year after the publication of the San Francisco Department of Public Health guidelines on Doxy-PEP^18^ and its use has been recently reported in MSM communities in China, despite being still limited to a small proportion of subjects (around 12%).^19^ Taken together, these data highlight how awareness and use of doxycycline for STI prophylaxis is spreading at the global level.

Interestingly, nearly half of those who referred Doxy-PEP/PrEP use in our survey acquired the antibiotic without formal medical oversight, in line with the observations of the Dutch survey that reported informal use of Doxy-PEP.^16^ This finding indicates a lack of structured access, guidance, and clinical supervision, especially when doxycycline is obtained outside formal healthcare settings. This raises concerns about unsupervised self-medication and suboptimal antibiotic use, which may increase the risk of resistance development and reduce treatment effectiveness.^20^

Noteworthy, clear guidelines are also needed for the clinicians. The IUSTI Europe Position Statement recommends identifying individuals with the highest risk for bacterial STIs, since they would most benefit from Doxy-PEP.^10^ Based on CDC clinical guidelines,^9^ around half of our MSM attendees should be counseled for Doxy-PEP (being MSM with a history of at least one STI among syphilis, chlamydia, or gonorrhea during the past year). However, a study conducted among HIV-PrEP users in Italy found that selecting MSM/TW for Doxy-PEP prescription based on the STI history in the last 12 months might lead to antibiotic overuse.^21^ On the other hand, sexual activities/behaviors may affect a possible recommendation of Doxy-PEP/PrEP. Indeed, the same CDC also state that Doxy-PEP could be discussed with MSM and TW who have not had a bacterial STI in the previous year but will be participating in sexual activities that may increase likelihood of exposure to STIs.^9^ UK guidelines include recent history (last 3 months) of multiple new, occasional, sexual partners, group-sex and chemsex as additional factors to be taken into account for the identification of individuals at elevated risk for syphilis acquisition.^11^

The observed association between doxycycline use and having had multiple sexual partners in the last year suggests that the individuals who are most likely to benefit from chemoprophylaxis are indeed those using it. These findings align with international studies indicating that Doxy-PEP users are often highly sexually active, frequently engage in group sex or condomless intercourse, and are likely to be on HIV-PrEP.^4,5^ While biomedical prevention strategies, such as those based on doxycycline use, are promising, their effectiveness and safety depend on integrated sexual health services that offer regular follow-up, behavioral counseling, and resistance monitoring.^22^

Despite the IUSTI Europe Position Statement,^10^ Italian clinicians currently operate in a context lacking national guidance on STI chemoprophylaxis. This uncertainty may contribute to the limited use as well as the low formal prescription rates observed in our survey. Concerns about cross-resistance certainly play a role, since studies have reported increasing tetracycline resistance in *Neisseria gonorrhoeae* and other microorganisms.^23,24^ European health authorities have rightly stressed the need for robust antimicrobial stewardship alongside any rollout of doxycycline-based prevention programs.

A few limitations of this study are recognizable. Firstly, this was a single center survey, which might not reflect the use of doxycycline prophylaxis in other MSM communities in Italy. Secondly, the number of Doxy-PrEP/PEP users was quite small, reducing our ability to identify other significant associations. Nonetheless, the still limited use of doxycycline prophylaxis, despite emerging from a single center survey, points to a clear opportunity. MSM already engaged in biomedical prevention, such as HIV-PrEP, may be particularly receptive to structured Doxy-PEP/PrEP interventions. Public health authorities should consider pilot programs that integrate these preventive strategies into existing HIV-PrEP services, supported by clinical monitoring, patient education, and resistance surveillance.

In conclusion, doxycycline remains an uncommon choice for STI prevention among our attendees and its usage is linked to individuals engaging in sex with multiple partners. Structured access, clinical supervision, and national guidelines are urgently needed to ensure that this promising strategy is both effective and safe.

## Data Availability

Research data is available on request from the corresponding author.*
